# Pericardial Effusion as a First Manifestation of Rheumatoid Arthritis: A Case Report

**DOI:** 10.7759/cureus.50489

**Published:** 2023-12-13

**Authors:** Fahad S Alqahtani, Yasser J Alashhab, Hanady O Manasfi, Wejdan Alanazi, Abdulaziz I Alsenani

**Affiliations:** 1 Internal Medicine, Prince Sultan Military Medical City, Riyadh, SAU; 2 Cardiology, Specialized Medical Center Hospital, Riyadh, SAU; 3 Rheumatology, Specialized Medical Center Hospital, Riyadh, SAU; 4 Internal Medicine, Specialized Medical Center Hospital, Riyadh, SAU

**Keywords:** rheumatology, rheumatoid arthritis, cardiology, pericardial effusion, pericarditis

## Abstract

Rheumatoid arthritis (RA) is a systemic autoimmune disease that mainly affects the joints, which can lead to joint deformity. Since the disease is systemic, it affects many organs, including the heart, which can lead to pericarditis, coronary artery disease, and heart failure. We are reporting on a male patient, 34 years of age and Sudanese, who complained of shortness of breath and chest pain that started weeks before he came to the hospital, with no other associated symptoms. The patient was admitted to the hospital, and extensive work was done for the patient, which revealed that he had pericardial effusion secondary to RA, which is the first presentation of the disease. RA rarely presents as a first presentation with pericarditis and pericardial effusion. The patient was managed medically, and he showed significant improvement.

## Introduction

Rheumatoid arthritis (RA) is a systemic autoimmune disease that mainly affects the joints, which can lead to joint deformity. Since the disease is systemic, it affects many organs, including the heart, which can lead to coronary artery disease, pericarditis, and heart failure [1]. The most common cardiac complication from RA is pericarditis, which accounts for almost 40% of cases and leads to fluid collection in the pericardium [1,2]. Most patients are asymptomatic; at most, 10% have the symptoms of pericarditis [3]. Additionally, if the anti-cyclic citrullinated peptide titer strands are elevated, the patient is at risk of developing pericardial effusion [4]. Sometimes, a patient can mask pericardial effusion symptoms when they are physically fit [4]. Pericarditis was associated with disease activity in RA [5]. In the United States, pericardial effusion affects nearly 6.5% of the general population. It can be present secondary to other diseases, such as infection, trauma, RA, systemic lupus erythematosus, and malignancy [6]. In this case, the patient had pericarditis and pericardial effusion as the first presentation of RA, which is a rare condition.

## Case presentation

**Figure 1:**
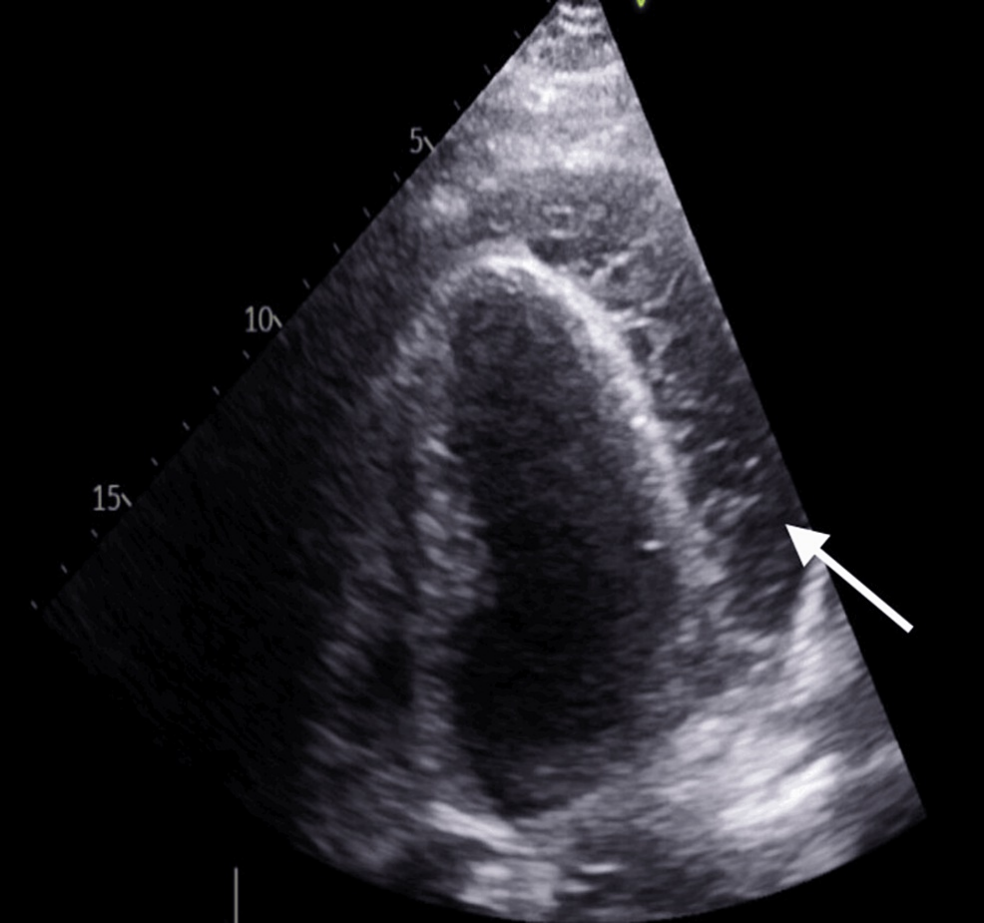
Trabeculated pericardial effusion

**Figure 2:**
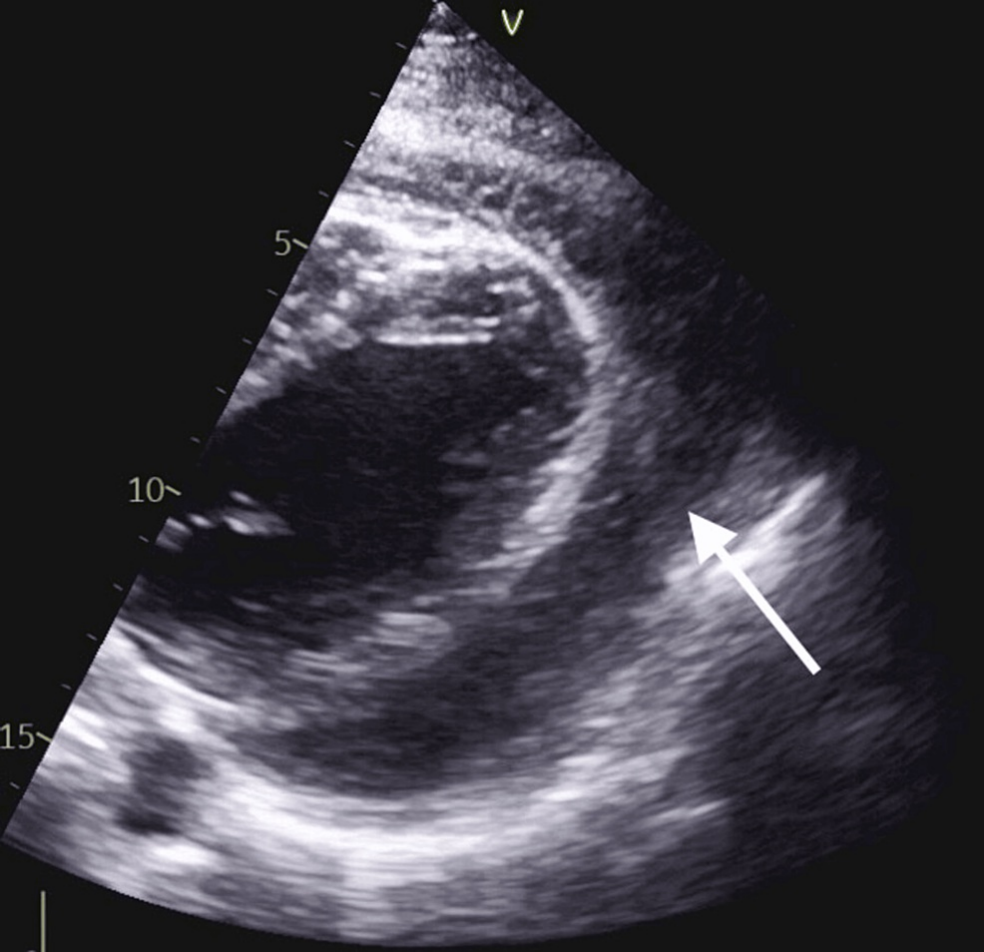
Trabeculated pericardial effusion

The 34-year-old Sudanese male, who was not known to have a chronic medical history, was referred from another hospital as a case of pericardial effusion and failed pericardiocentesis without reaching any diagnosis. The patient complained of shortness of breath and chest pain that started three weeks before his first admission, with no aggravating or relieving factors. In addition, the patient reported new mild left knee pain with no morning stiffness while admitted to the hospital. The patient denied any history of fever, cough, syncope, nausea, vomiting, diarrhea, skin rash, recent surgery, and recent travel. Upon presentation, the patient had a temperature of 36.8 °C, a heart rate of 107 beats per minute, a respiratory rate of 20 breaths per minute, a blood pressure of 132/88 mmHg, and an oxygen saturation of 98% in room air. He was conscious, oriented, and alert with a Glasgow coma scale of 15/15; he was not in distress, and there was no cyanosis or clubbing. His chest examination was clear to auscultation and percussion, and a CVS examination revealed normal heart sounds, presenting S1 and S2 with a normal rate and regular rhythm. An abdominal examination revealed a soft and lax abdomen with no tenderness or organomegaly. He had mild left knee swelling with no lower limb edema, redness, or hotness. The laboratory work (Table 1) also revealed that his kidney function, liver function, and enzymes were normal. Electrocardiography showed sinus tachycardia, and the chest X-ray was unremarkable. A computed tomography pulmonary angiogram was done, and pulmonary embolism was ruled out; an echocardiogram showed a large, highly trabeculated pericardial effusion (Figures 1-2). The patient was diagnosed with RA complicated with pericardial effusion. The cardiology and rheumatology teams met and decided to treat the pericardial effusion medically without the need for an intervention. The patient began taking colchicine 0.5 mg twice a day and ibuprofen 400 mg three times a day; the patient showed improved symptoms and was discharged after four days of admission. In the outpatient department, he followed up with the Cardiology Department, and another echocardiography was done after 10 days, which showed a significant regression of the effusion. The patient also followed up with rheumatology and began taking hydroxychloroquine 400 mg daily.

**Table 1:** Laboratory workup

Investigation	Result	Reference range
White blood cell count	12.70 K/UL	4.5-11.0 K/UL
Hemoglobin	14.60 g/dL	13.5-17.5 g/dL
Platelet count	425 10S3/UL	150-450 10S3/UL
PRO-BNP	172 pg/mL	0-125 pg/mL
Serial troponin	Normal	-
Pro-calcitonin	0.11 ng/mL	0-0.5 ng/mL
ESR	75.000 mm/hr (elevated)	Depending on the age and gender
CRP	12.6 mg/dL	0-0.5 mg/dL
Thyroid-stimulating hormone	1.3 uIU/mL	0.270-4.2 uIU/mL
Ferritin	637 ng/mL	30-400 ng/mL
Antinuclear antibody screen IgG	Negative	Negative
Complement C3	195 mg/dL	90-180 mg/dL
Complement C4	33 mg/dL	10-40 mg/dL
Anti-double-stranded DNA IgG	Negative	Negative
Rheumatoid factor	120.5 IU/mL	10-14 IU/mL
ANTI-CCP	89.86 U/mL	7-17 U/mL
Antistreptolysin O antibody	62 IU/mL	20-200 IU/mL

## Discussion

Pericarditis is a well-known extra-articular manifestation of RA. However, it is unusual for pericarditis to present in undiagnosed RA and as the first presentation of the disease. Few reported cases are similar to this patient’s. RA can cause a variety of cardiac manifestations, although pericarditis is the most common [1]. Even if the patient does not complain of typical joint pain, RA cannot be ruled out [6]. Moreover, other causes, including malignancy, infectious causes, and hypothyroidism, must be ruled out [3]. Aspirin and NSAIDs are the cornerstones of treating pericarditis [2]. After this patient began NSAIDs and colchicine, he showed significant improvement. Conversely, another study has shown that, after starting the patient on diclofenac (75 mg/day) and colchicine (1.2 mg/day), the patient did not improve and developed cardiac tamponade [5]. It is essential to start the patient with the appropriate dose of NSAIDs and colchicine [2]. Pericardiocentesis is part of the diagnostic workup [6], which is unnecessary if we have an apparent reason for the pericardial effusion. It is essential to do this when the patient has developed cardiac compression symptoms, such as lower limb pitting edema, elevated jugular vein pressure [7], or developed cardiac tamponade [5].

## Conclusions

We reported an unusual presentation of RA in which the patient presented with shortness of breath and chest pain. Extensive workup was done for the patient, and he was diagnosed with RA complicated with pericardial effusion. The patient did not have typical joint pain from RA. The patient underwent pericardiocentesis in another hospital, which failed. Then, the patient was treated medically with NSAIDs and colchicine and showed significant improvement. This case highlights the importance of recognizing pericardial effusion as a potential sign of undiagnosed RA and the need for further investigation.
